# Effect of clonidine as adjuvant in bupivacaine-induced supraclavicular brachial plexus block: A randomized controlled trial

**DOI:** 10.4103/0253-7613.64498

**Published:** 2010-04

**Authors:** Susmita Chakraborty, Jayanta Chakrabarti, Mohan Chandra Mandal, Avijit Hazra, Sabyasachi Das

**Affiliations:** 1,4 Department of Pharmacology, Institute of Postgraduate Medical Education & Research, Kolkata, India; 2 Department of Anaesthesiology, Bangur Institute of Neurosciences & Psychiatry, Kolkata, India; 3,5 Department of Anaesthesiology, North Bengal Medical College and Hospital, Sushrutanagar, Darjeeling, India

**Keywords:** Clonidine, bupivacaine, supraclavicular brachial plexus block, adjuvant, duration of analgesia, randomized controlled trial

## Abstract

**Objective:**

Clonidine has been used as adjuvant to local anesthetics in order to extend the duration of analgesia in various regional and central neuraxial blocks. It is previously reported that clonidine added to bupivacaine increases analgesia duration in brachial plexus block. We evaluated the effect of this combination in supraclavicular brachial plexus block for upper limb orthopedic procedures.

**Materials and Methods:**

A randomized double-blind placebo controlled trial was done with 70 patients of American Society of Anesthesiologists Grade I or II status undergoing upper limb orthopedic procedures. Group A (n = 35) patients received 25 ml of 0.5% bupivacaine and 0.2 ml (30 mcg) clonidine, whereas group B (n = 35) received 25 ml of 0.5% bupivacaine and 0.2 ml normal saline through a supraclavicular approach for brachial plexus block. Vital parameters were recorded 10 min prior to block placement and every 3 min thereafter till the end of the procedure. Onset and duration of both sensory and motor blocks and sedation score were recorded. All patients were observed in postanesthesia care unit and received tramadol injection as soon as they complained of pain as rescue analgesic. Duration of analgesia was taken as the time from placement of block till injection of rescue analgesic.

**Results:**

Analgesia duration was 415.4 ± 38.18 min (mean ± standard deviation) in Group A (clonidine) compared to 194.2 ± 28.74 min in Group B (control). No clinically significant difference was observed in heart rate, blood pressure, and oxygen saturation. Sedation score was higher in the clonidine group.

**Conclusion:**

Addition of a small dose of clonidine to 0.5% bupivacaine significantly prolonged the duration of analgesia without producing any clinically important adverse reactions other than sedation.

## Introduction

Acute postoperative pain is the result of a complex physiological reaction to tissue injury. The dorsal horn of the spinal cord is the site of termination of primary afferents and there is complex interaction between such afferent fibers, intrinsic spinal neurons, descending pain modulating fibers, and various associated neurotransmitters such as serotonin, norepinephrine, acetylcholine, adenosine, and glutamate in the dorsal horn.[1] Local anesthetics administered as regional nerve blocks are utilized in providing postoperative pain relief in many surgical procedures by blocking signal traffic to the dorsal horn. Certain drugs may be used as adjuvant to local anesthetics to lower doses of each agent and enhance analgesic efficacy while reducing the incidence of adverse reactions. Tramadol and fentanyl had been successfully used as adjuvants to local anesthetic in brachial plexus block.[2,3] The concurrent injection of 2 adrenergic agonist drugs has been suggested to improve the nerve block characteristic of local anesthetic solutions through either local vasoconstriction[4] and facilitation of C fiber blockade[5] or a spinal action caused by slow retrograde axonal transport or simple diffusion along the nerve.[6] Clonidine is a selective 2 adrenergic agonist with some 1 agonist property. In clinical studies, the addition of clonidine to local anesthetic solutions improved peripheral nerve blocks by reducing the onset time, improving the efficacy of the block during surgery and extending postoperative analgesia.[7,8] The effect of clonidine is dose related between 0.1 and 0.5 *μ*g/kg.[8] Clonidine possibly enhances or amplifies the sodium channel blockade action of local anesthetics by opening up the potassium channels resulting in membrane hyperpolarization, a state in which the cell is unresponsive to excitatory input.[9]

A number of these studies have focused on the effect of clonidine as adjuvant to either lignocaine[8] or mepivacaine.[7] Further, these studies were done using clonidine 150 mcg, a moderately high dose with its attendant risk of adverse drug reactions. In a few clinical studies, a lower dose of clonidine (0.1-0.5 mcg/kg) was used as adjuvant for brachial plexus block.[7] Considering the fact that Indian population has relatively lower body weight and that there are few studies with low dose clonidine, we planned to compare the effect of low dose clonidine versus placebo as adjuvant to bupivacaine for brachial plexus block, by supraclavicular approach, for orthopedic procedures of moderate duration.

## Materials and Methods

### Study Setting and Participants

The study was conducted in tertiary care teaching hospital between December, 2007 and September, 2008. Written informed consent was obtained from all patients and the study was approved by the Institutional Ethics Committee.

Seventy patients aged 18 to 60 years, scheduled for elective orthopedic operations in the upper limb, under supraclavicular brachial plexus block, were included in this study. They were of American Society of Anesthesiologists (ASA) Grade I or II physical status. The procedures were of moderate duration and included implant removal, both bone plating, fixation of lower third of humerus and olecranon fixation. Patients receiving chronic analgesic therapy, those with severe cardiopulmonary disease, thyroid disorders, diabetes mellitus, central or peripheral neuropathies, history of allergy to local anesthetics, or other contraindications to regional anesthesia were excluded from the study.

### Randomization and Blinding

The study was designed as a prospective, randomized, double-blind, placebo-controlled trial. Participants were allocated to two equal groups of 35 each using a computer generated random number list. Group A patients received 25 ml of 0.5% bupivacaine and 0.2 ml (30 mcg) clonidine, while group B received 25 ml of 0.5% bupivacaine and 0.2 ml of 0.9% sodium chloride through a supraclavicular approach for brachial plexus block. The allocation sequence was generated by the author entrusted with statistical analysis. The anesthesiologist administering the injections and observing the effects received serially numbered sealed envelopes indicating the A or B codes for the anesthetic mixture to be administered. The A and B syringes were loaded with drug by another author not involved in administering the injections and in further evaluation of the patients. All observations (hemodynamic variables, oxygen saturation, level of sedation, time required to achieve surgical block in the operation theater and the time to rescue analgesic in the postanesthesia care unit) were also recorded in a blinded manner.

### Interventions

Once a patient was brought into the operation theater, standard monitoring was set up, including noninvasive arterial blood pressure, heart rate, and pulse oximetry. An 18-gauge IV cannula was inserted in the forearm and an infusion started with lactated Ringer's solution. Midazolam 0.05 mg/kg IV bolus was used for sedation[10] after the block was achieved, so as to allay apprehension and anxiety during the operative procedure. The surgical procedure was performed by using a standard arm tourniquet inflated to 70 mmHg higher than systolic blood pressure. Hemodynamic variables were measured 10 min before block placement and every 3 min thereafter till the end of surgery.

Nerve blocks were performed, with the aid of a nerve stimulator, by using a 22G short-beveled, insulated (Teflon®-coated) 25 mm long stimulating needle. Stimulation frequency was set at 2 Hz, while the intensity of stimulating current was initially set to deliver 1 mA and gradually decreased to < 0.5 mA. Negative aspiration was performed while injecting the drug solution to avoid any intravascular placement. Sensory and motor blocks on the operated limb were evaluated at 2, 5, 10, 20, 30 and 60 min after the completion of anaesthetic injection by one of the authors who were unaware of the drug combination administered. Sensory block was assessed by pinprick discrimination (with 22G hypodermic needle) and motor block was evaluated by asking the patient to move the forearm against resistance and to flex the forearm. A pinprick sensation on the contralateral arm was scored as 100 points. Patients were requested to compare pinpricks in the primary innervation areas of the respective nerves in the anesthetized arm with the contralateral arm as reference. The scale ranged from 100 points (full sensation) to 0 points (no sensation). Brachial plexus block was considered successful by Vester-Andersen's criteria[11] when at least two out of four nerve territories (radial, ulnar, median, and musculocutaneous) were effectively blocked. Onset of sensory block was defined as a reduction of sensibility to 30% or less while onset of motor block was defined as reduction of muscle power to grade 3 or less. The time to surgical blockade was defined as the time from the end of anesthetic injection to loss of pinprick sensation along the distribution of the ulnar and radial nerves along with inability to circumrotate the thumb of the concerned limb. When surgical anesthesia was not achieved in a patient even after 30 min from the anesthetic injection, the case was considered as failed block and the operation was then performed under general anesthesia.

Following operation, all patients were observed in postanesthesia care unit and received rescue analgesic as soon as they complained of any pain. This consisted of tramadol 100 mg IV, repeated if necessary. Patients were given clear instruction to ask for a rescue analgesic as soon as they sensed discomfort caused by pain on the operated hand. The time from the end of anesthetic injection in the operated hand till the first request for postoperative rescue analgesic was recorded in each patient.

### Assessments

The primary outcome measure was duration of analgesia. This was estimated as the time interval from placement of the block till first injection of rescue analgesic. Secondary outcome measures were onset and duration of sensory and motor blockade and any suspected adverse drug reactions.

Noninvasive arterial blood pressure, heart rate and hemoglobin oxygen saturation monitoring was done throughout the procedure. Clinically relevant bradycardia (heart rate < 45 bpm) spells were treated with atropine (0.6 mg IV) and their occurrence was recorded. The degree of sedation was evaluated by using the University of Michigan Sedation Scale (UMSS)[12] of 0 to 4 [0 = awake and alert; 1 = minimally sedated/sleepy, appropriate response to conversion and/or sound; 2 = moderately sedated, somnolent/sleepy, easily aroused with tactile stimulation and/or simple verbal command; 3 = deeply sedated/deep sleep, aroused only with significant stimulation and 4 = could not be aroused].

All patients were clinically assessed during discharge from the orthopedic ward and again after 3 weeks (at the first routine postoperative examination) for occurrence of any neurological complications.

All 35 patients in the two groups were considered for adverse event analysis. However, subjects who failed blocks were excluded from effectiveness assessment.

### Sample Size and Statistical Analysis

Duration of analgesia was taken as the outcome measure of interest for the purpose of sample size calculation. It was estimated that 23 subjects would be required per group in order to detect a difference of 30 min in this parameter between the two groups, with 90% power and 5% probability of Type 1 error. This calculation assumed a pooled standard deviation of 30 min for the duration of analgesia.

Data are summarized as mean ± standard deviation or as percentages. Statistical analysis was performed by Statistica version 6 [StatSoft Inc.; Tulsa, Oklahoma, USA; 2001] and GraphPad Prism version 4 [GraphPad Software Inc.; San Diego, California, USA; 2005] software. Comparison of categorical variables between the two groups was by Chi-square test or Fisher's exact test, as appropriate. Numerical variables were normally distributed and were compared by Student's unpaired 't'-test. All analyses were two-tailed and *P* < 0.05 was considered statistically significant.

## Results

We recruited 35 subjects per group, more than the calculated sample size. There were no dropouts. However, excluding subjects who failed blocks, 32 patients in the clonidine group and 31 in the normal saline group were eligible for effectiveness analysis. The difference in the number of valid blocks in the two groups was not statistically significant.

The age, sex distribution, body weight, and duration of surgery in the two groups were found to be comparable [Table 1]. Onset and duration of sensory and motor blocks have been presented in Table 2. It was found that onsets of both sensory and motor block were significantly shorter whereas durations were significantly greater in the group receiving clonidine.

**Table 1:** Comparison of demographic and other relevant parameters at baseline between the two groups

*Baseline characteristic*	Bupivacaine + clonidine (n = 32)*	Bupivacaine + saline (n = 31)*	*P value*
Age (years)	41.65 ± 13.26	41.70 ± 9.54	0.985
Sex(Male : Female)	22 : 10	21 : 10	1.000
Height (cm)	152.4 ± 11.15	152.0 ± 10.87	0.781
Weight (kg)	55.9 ± 6.81	57.1 ± 7.52	0.501
Duration of orthopedic procedure (min)	105.6 ± 13.13	109.2 ± 13.98	0.292

* Here n denotes the number of subjects with valid blocks out of 35 patients in each group, Values of numerical variables have been expressed as Mean ± standard deviation.

**Table 2:** Time profiles of sensory and motor blocks and duration of analgesia in the study groups

*Baseline characteristic*	Bupivacaine + clonidine (n = 32)*	Bupivacaine + saline (n = 31)*	*P value*
Onset of motorblock (min)	10.6 ± 1.36	18.1 ± 1.35	< 0.001
Duration of motor block (min)	330.4 ± 31.68	144.8 ± 17.31	< 0.001
Onset of sensory block (min)	6.2 ± 0.78	8.7 ± 1.01	< 0.001
Duration of sensory block (min)	279.1 ± 28.98	116.0 ± 17.16	< 0.001
Onset of surgical block	13.4±1.84	20,3±1.91	< 0.001
Duration of analgesia (min)	415.4 ± 38.18	194.2 ± 28.74	< 0.001

* Here n denotes the number of subjects with valid blocks out of 35 patients in each group,Values of numerical variables have been expressed as Mean ± standard deviation.

Regarding time to onset of surgical block, this was also faster by about 7 min [Table 2] in the clonidine adjuvant group.

The mean time from block placement to first request for pain medication i.e. the duration of analgesia was 415.4 ± 38.19 min in the clonidine group but 194.2 ± 28.74 min in the other group. This difference (about 221 min) was highly significant (*P* < 0.001) statistically as well as clinically.

Although patients who received clonidine were found to be more sedated in comparison to those who did not [Table 3], no statistically significant difference was observed in heart rate, blood pressure, and oxygen saturation between the two groups at any time point. The incidence of clinically relevant bradycardia and hypotension were comparable between groups. Only one patient receiving clonidine complained of restriction of movement in the operated arm during postoperative visit after 3 weeks, which was found to be clinically trivial.

**Table 3:** Suspected adverse drug reaction profile in the two study groups

*Suspected adverse reaction*	*Bupivacaine + clonidine (n = 35)*	*Bupivacaine + saline(n = 35)*	*P value*
Bradycardia (heart rate < 45bpm)	1	0	1.000
Hypotension (fall in mean arterial pressure > 20% of base line)	8	6	0.766
Oxygen saturation < 90%	0	0	
Sedation score (mean ± standard deviation)	2.4 ± 0.75	1.7 ± 0.53	< 0.001
Postoperative arm weakness	1	0	1.000

*At post operative visit after 3 weeks

## Discussion

The result of the present randomized controlled trial clearly suggests that relatively low-dose clonidine, as adjuvant to 0.5% bupivacaine for supraclavicular brachial plexus block, prolongs the duration of analgesia as well as motor block. Onset times of blocks were also shown to be shortened though the study was not powered to measure these effects. These findings are at variance with the study by Duma *et al* which showed no difference in analgesia after addition of clonidine 0.5 *μ*g/kg to levobupivacaine in axillary block.[13] Probable explanation for this inconsistency may relate to inter-patient variations in the anatomy of the plexus sheath and difference in the spread of local anesthetics in the plexus sheath depending upon the block technique. More explanations may be forthcoming when the mechanism of adjuvant action of clonidine in this setting is elucidated.

Bernard and Macarie,[8] evaluating the effects of adding 30-300 *μ*g clonidine to lignocaine for axillary brachial plexus anesthesia, reported that the addition hastened the onset of the block and improved the efficacy of surgical anesthesia. There are reported differences in the effects of administration of low-dose clonidine on time of onset and efficacy of nerve block, which may be explained by differences in the type of nerve block, exact mixture injected, and technique used to perform the block (single injection versus multiple injections). In fact, a multiple-injection technique was used, which is known to improve both onset time and quality of nerve block,[14] and this could have reduced the differences in onset time between the two groups.

In a dose-finding study evaluating the minimum effective dose of clonidine required to prolong duration of analgesia after axillary brachial plexus block, Singelyn *et al*.[7] suggested that 0.5 *μ*g/kg clonidine should be used. At this dose, significant prolongation of analgesia was achieved without undue sedation, hypotension, or bradycardia. It has been widely demonstrated in different studies that subcutaneous or intramuscular injection of clonidine is not as effective as perineural administration,[15] suggesting that the local anesthetic-prolonging effect of clonidine is probably mediated locally at the neuron.[16] This may also explain the variation in response in different types of peripheral nerve blocks, probably related to the rate and extent to which the injected anesthetic solutions penetrate into the nerve.[11] Even though injecting clonidine as the sole analgesic into the brachial plexus sheath does not provide clinically relevant analgesia,[17] it has been demonstrated to inhibit the action potential of A and C fibers in de-sheathed sciatic nerves.[9] Many authors favor the hypothesis that clonidine exerts its local anesthetic-prolonging effect directly on the nerve fiber, as a result of complex interaction between clonidine and axonal ion channels or receptors.[5,11,15] Peripheral antinociception induced by clonidine has also been related to 2-adrenoceptor-mediated local release of enkephalin-like substances.[18]

We selected a 30 *μ*g dose of clonidine keeping in mind the hemodynamic adversities that might be produced. It was found that this dose provided satisfactory prolongation of the duration of analgesia without producing significant hemodynamic compromise in the patients. However, clonidine did induce greater sedation in the patients during the early part of their stay in postanesthesia care unit. Therefore, we cannot say that this is the ideal dose of clonidine as adjuvant to 0.5% bupivacaine for supraclavicular brachial plexus block. This conclusion can only be drawn after a definitive dose finding study.

Keeping this limitation in mind, we can suggest that 30 *μ*g of clonidine may be used as an adjuvant to 0.5% bupivacaine for supraclavicular brachial plexus block so as to prolong postoperative analgesia without added problems apart from some sedation in the early postoperative period.
